# Reliable mortality statistics in Myanmar: a qualitative assessment of challenges in two townships

**DOI:** 10.1186/s12889-019-6671-y

**Published:** 2019-03-29

**Authors:** Myitzu Tin Oung, Kerry Richter, Pramote Prasartkul, Yadanar Aung, Kyaw Thu Soe, Thuzar Chit Tin, Viroj Tangcharoensathien

**Affiliations:** 1 Institute for Population and Social Research, Mahidol University, Bangkok, Thailand; 2 Department of Medical Research (Pyin Oo Lwin Branch), Pyin Oo Lwin, Myanmar; 3 Department of Public Health, Shan State, Myanmar; 4 International Health Policy Program, Ministry of Public Health, Bangkok, Thailand

**Keywords:** Mortality statistics, Vital registration system, Myanmar

## Abstract

**Background:**

The vital registration system is universally recognized as the main source of mortality data which is essential for policy formulation, proper interventions and resource allocation to address priority health challenges. To improve availability and quality of mortality statistics by strengthening the vital registration system, understanding the current vital registration system is essential. This study identified challenges in generating reliable mortality statistics in the vital registration system of Myanmar.

**Methods:**

Qualitative methods were used to collect data in two selected townships of Mandalay Region. Grey literature related to the management of mortality registration was reviewed; in-depth interviews of sixteen key informants and fourteen focus group discussions were conducted with those involved in death registration at the local level, such as healthcare providers, local administrators and knowledgeable adults in households where deaths occurred during the past three years. Thematic analysis was performed to identify system barriers in the death registration process.

**Results:**

Weaknesses in the death registration system are classified in three areas: a) administrative which includes the lack of enforcement of mandatory death registration, limited issuance of death certificates and no formal mandatory notification of death events by households and; b) technical which includes absence of proper and regular on-the-job trainings, ineffective cause-of-death certification practice for deaths in the communities and the absence of routine data plausibility checks at the local level; and c) societal which includes poor community awareness and inadequate participation in death registration.

**Conclusion:**

The study highlighted challenges in the death registration system at the operational level, which undermines the achievement of a satisfactory level of completeness and accuracy of mortality data. We recommend establishing a strong legal framework, improving technical capacities and raising public awareness and cooperation to strengthen the system that can generate reliable mortality statistics.

**Electronic supplementary material:**

The online version of this article (10.1186/s12889-019-6671-y) contains supplementary material, which is available to authorized users.

## Background

Evidence-informed health policies and interventions contribute to improving the health of populations. Among others, reliable and timely mortality statistics are essential for policy formulation, proper interventions and resource allocation to address priority health challenges. The vital registration system (VRS) is universally recognized as the main source of mortality data as it generates mortality profiles of a population on a continuous basis for time trend monitoring. Despite its importance, mortality statistics in developing countries are often inadequate [1].

**Fig. 1:**
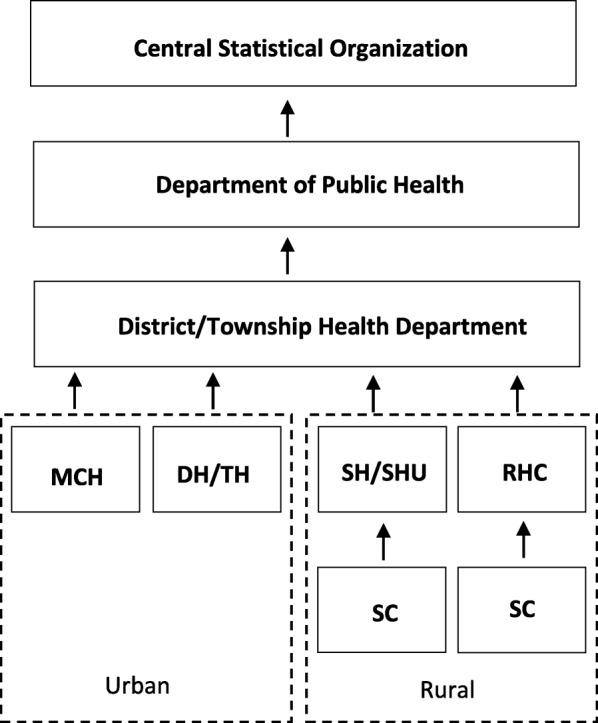
Formal reporting process of vital information in the VRS of Myanmar. MCH: Maternal and Child Health Center; DH/TH: District/Township Hospital; SH: Station Hospital; SHU: Station Health Unit; RHC: Rural Health Center; SC: Sub-Health Center

In Myanmar, registration of vital events was started in some parts of lower Myanmar in 1904 and extended to upper Myanmar in 1906 [2]. Only after the Myanmar-UNICEF Country Programme, 2001–2005, has the VRS run uniformly throughout the country by applying eight standardized forms. Of these, five forms are used for death registration (Table 1) [3]. The Central Statistical Organization (CSO) is the responsible agency for the national system of statistical information while the Department of Public Health (DoPH) and Department of Medical Services (DMS) are the implementing agencies [4]. Local public health staff and hospital staff are mandated to register and record vital events, issue certificates to families and report to the CSO (Fig. 1). There are more challenges in death registration than in birth registration to reach a satisfactory level of completeness and accuracy because of the lack of laws to enforce mandatory registration of deaths, unequal access to services, no perceived benefits for registering deaths, low level of community awareness, and resource limitations [5]. The country barely conducted research which investigated the framework and operations of death registration in the VRS.

**Table 1:** Standardized forms used in the death registration in Myanmar

Form number	Form name	Action taken
Form 201^a^	Death Record	Send to CSO
Form 202	Death Register	Keep in the health facility
Form 153	Still-birth Certificate	Issue to the family on request
Form 203	Death Certificate	Issue to the family on request
Form 204 (includes still-birth)	Burial Certificate	Issue to the family on request

^a^For hospital deaths, “Medical Certificates of Cause of Death” form has to be attached with Form 201

It is essential to reach a high level completeness of registration (90% or higher) to generate reliable mortality statistics; if this is not possible, a minimum of 60% completeness is needed to produce useable mortality data by applying demographic techniques [1]. However, completeness of death registration in Myanmar VRS has been estimated at less than 50% [6]. A recent assessment of the vital statistics systems of 148 countries was performed using the Vital Statistics Performance Index (VSPI), which measures quality of COD reporting, quality of age and sex reporting, internal consistency, completeness of death reporting, the level of cause-specific detail and data availability/timeliness. The lower the score, the poorer the VRS performance. The findings showed Myanmar with a lower VSPI score (0.018), compared with neighboring countries such as Malaysia (0.75) and Thailand (0.57) [7].

To improve the quality of mortality statistics, understanding the current performance of the vital registration system is essential. The objective of this study was to assess performance barriers of the Myanmar vital registration system, in terms of administrative challenges, lack of technical capacities and public awareness and cooperation [8, 9]. This study may contribute to policy and administrative reform.

## Methods

### Study design

Qualitative methods were used to obtain information, including reviews of relevant grey literature related to operating procedures of death registration (such as manuals and protocols), Key Informant Interviews (KII) and Focus Group Discussions (FGD).

### Study period

The study was conducted during the period of January and August, 2016.

### Study area

Information was collected from two selected townships of Mandalay Region which has a population of about 6.2 million (2.2 million in urban areas and 4.0 million in rural areas), residing in 28 townships. The selection of two townships emphasized understanding how death registration practices differ between the two different contexts, i.e. more urbanized Myingyan Township with 31.8% urban population and the less urbanized Myittha Township with 9.9% urban population. The reasons for selecting study areas were: 1) The procedures for death registration and reporting practices in the two townships could reflect the procedures established in the country as the VRS has been uniformly implemented in all states and regions since 2009; 2) Accessibility to the two townships was feasible for the researcher to conduct the field work with limited funding.

### Study population

The study participants were 1) healthcare providers including medical officers (MO), health assistants (HA), lady health visitors (LHV) and midwives (MW) who were responsible for reporting all births and deaths; 2) local administrators such as Ward Administrator (WA) or Village Tract Administrators (VTA) who were also playing some roles in vital registration; 3) knowledgeable adults in households where deaths occurred during past three years representing the views and practices in the communities.

### Data collection

**Fig. 2:**
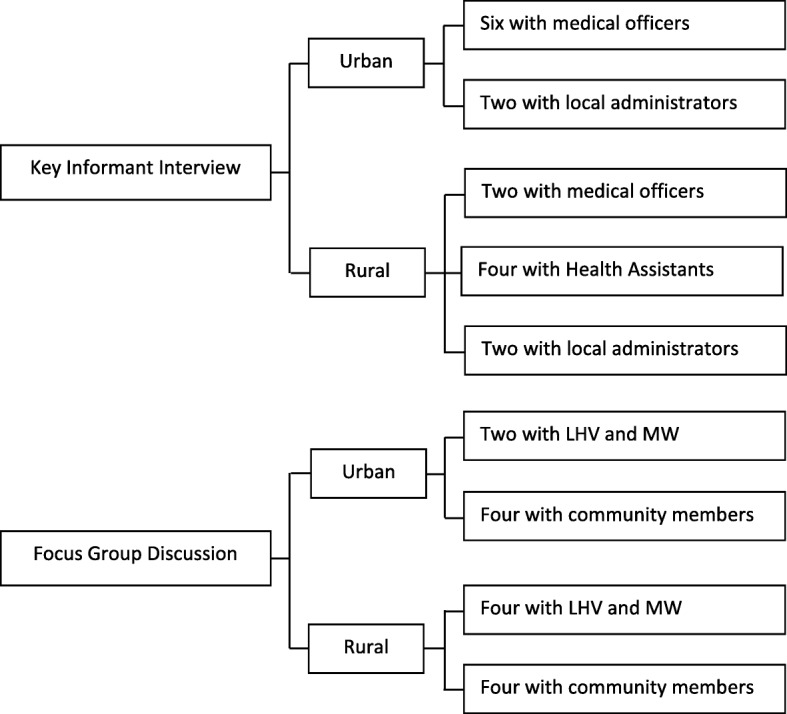
Number of Key Informant Interviews (KII) and Focus Group Discussions (FGD) in two townships. Note: LHV: Lady Health Visitors; MW: Midwives

Sixteen KIIs (8 in urban and 8 in rural) and fourteen FGDs (6 in urban and 8 in rural) were conducted in two selected townships (Fig. 2), as information from these KIIs and FGDs became exhaustive and was adequate to meet the study objectives.

The government officials responsible for VRS and community member residing in urban and rural areas were selected for KII and FGD to understand the difference in awareness and cooperation in death reporting. The total number of respondents involved in both KII and FGD were 111 (16 Key informants and 95 FGD participants (Table 2).

**Table 2:** Respondent profiles: Key Informant Interviews (KII) and Focus Group Discussion (FGD)

Data collection method	Study population	Total no. of respondents	Residence		Mean age (in years)^a^	Gender		Mean service (in years)^a^
			Urban	Rural		Male	Female	
FGD	MW and LHV	40	14	26	35 (23–50)	–	40	10.7 (1–30)
FGD	Community members	55	26	29	49 (27–70)	21	34	–
KII	Local administrator	4	2	2	42 (28–59)	4	–	5.5 (1–8)
KII	MO	8	6	2	36 (30–44)	4	4	9.0 (3–16)
KII	HA	4	–	4	40 (31–49)	2	2	10.5 (3–20)
Total	–	111	48	63	–	31	80	–

^a^Figures in bracket are minimum and maximum

*FGD* Focus Group Discussion, *KII* Key Informant Interview, *MW* Midwife, *LHV* Lady Health Visitor, *MO* Medical Officer, *HA* Health Assistant

### Key informant interview (KII)

KIIs were conducted with those who are taking responsibility for or are involved in the VRS in the areas, such as MOs, HAs and local administrators, using KII guides (Additional files 1 and 2). The issues addressed in the KII were operational procedures of registering, recording and reporting of death events; their roles, responsibilities in the VRS; trainings received; cause-of-death certification practice; quality assurance procedures; and challenges in the VRS. Key informants were purposively selected based on their knowledge and experience, availability and willingness to participate. The study covered 8 MOs from the two Township Hospitals and two randomly selected Station Hospitals; 4 HAs from four randomly selected Rural Health Centers (RHCs); and 2 WA and 2 VTA from four randomly selected wards and villages.

### Focus group discussion

Fourteen FGDs were conducted, and five to nine respondents were participated in each FGD session. Six FGD (2 in urban and 4 in rural areas) were conducted with MWs and LHVs, who are responsible for vital registration at the community level, using FGD guides (Additional files 3 and 4). The respondents were from two Maternal and Child Health Clinics (MCH) in urban areas and from four selected RHCs in rural areas. The discussions explored the respondents’ functions in the VRS; how they registered and recorded deaths; and problems and challenges they encountered in the process.

Eight FGDs were conducted with community members, who experienced death events in households during past 3 years, in order to assess their awareness, knowledge and practice of death registration. The participants who can provide adequate information were recruited with the help of village volunteers and healthcare providers.

The guides were developed by the research team based on literature review and information obtained from informal interviews with health staff. Then, guides were pre-tested and finalized. KIIs and FGDs were conducted by the principal investigator and supported by two experienced co-investigators. All interviews and discussions were conducted in local language at a privacy place where respondents feel comfortable. Note taking and audio recording of interviews and focus group discussions were done by trained research assistants with the informed consent of the respondents.

### Data analysis

The records were verbatim transcribed in local language by the research team. Transcriptions were coded and organized for each subgroup of study participants. Then, thematic analysis of qualitative data was performed by the researchers using a qualitative data analysis software. Three main themes and seven sub-themes were identified by this study. Direct quotes were used for exemplary purposes. Confidentiality was fully adhered to.

## Results

The study identifies three main themes which were found to be the key barriers in managing high performance death registration at the local level: administrative challenges, technical capacities and societal contexts. Seven sub-themes were identified under main themes. In Table 2, the profiles of KII and FGD participants are described.

### The first barrier: administrative challenges

#### Lack of enforcement of mandatory death registration

Both document review and KII respondents indicated that there is no specific law enforcing mandatory death registration in Myanmar, though there are some related laws that are used to manage death registration, such as The City of Yangon Municipal Act, 1922; The Myanmar Village Headman Manual, 1948; The Development Affairs Act, 1993 and the Ward and Village Tract Administration Law, 2012 [3]. The Ward and Village Tract Administration Law (2012) is the most updated one, where every birth and death has to be reported to Ward Administrator (WA) or Village Tract Administrator (VTA) within three days (or 24 h if the death occurs due to infectious disease). Non-compliance should result in imprisonment of not exceeding seven days by the relevant court or a fine not exceeding fifty thousand kyats (approximately $38) [10]. However, according to Key Informant Interviews with local administrators, enforcement for non-compliance is rare, especially in rural areas.

WA and VTA whom referred to by the Ward and Village Track Administration Law are working under the Township General Administrative Office (TGAO) which is under General Administrative Department (GAD), Ministry of Home Affairs. The TGAO performs various other functions of government including recording and reporting of births and deaths to GAD. WA and VTA play roles in administration at the ward and village tract level. WA and VTA are supervised by Township Administrator and Deputy Township Administrator; supported and assisted by clerks and community leaders who are volunteers. In every ward and village tract, there are many community leaders who are assigned to every 10 households, 100 households, or a defined territory. They help WA or WTA in performing their functions by directly contacting with the households and communities [11].

The regulations related to vital registration functions of WA or the VTA are: 1) the WA and VTA have to provide instructions to the households for registering births and deaths within 3 days of occurrence of the event; 2) they have to supervise recording births and deaths and informing to health centers [10].

Although the law instructed to report every birth and death to WA or VTA, in the Vital Registration Training Manual, it was stated that, for vital events occurred in the community, death records (Form 201) and death register (Form 202) have to be completed by the Midwives (MW) or Public Health Assistant 2 (PHS 2). For events occurred at hospitals, the registered doctors has to complete the records including “Medical certificates of cause-of-death form”. Registering and reporting vital events to CSO are performed by health staff and supervised by the in charge of the health facility [3, 12].

#### Limited issuance of death certificates

District or Township Medical Officer has to take responsibilities to sign and issue Death Certificate (Form 203) and Burial Certificate (Form 204) to family members of the deceased upon request [3, 12]. The township medical officer (TMO) from KII explained that issuing death (Form 203) or burial certificates (Form 204) to the deceased’s family was not a routine procedure in the study township. It means, in general, people in the study areas do not request the certificates because of its limited usefulness. A death certificate is required to apply for a family pension if the deceased is a government official, to claim an inheritance, or to gain a burial certificate as required in some areas.

As confirmed by findings from FGDs and KIIs, a death or burial certificate is not necessarily required in rural areas. For cremation or burial in urban areas of the less urbanized township, a burial certificate from the township office of the General Administrative Department (GAD) or township Developmental Affairs Office (DAO), but not from the health center, is required. On the other hand, in the more urbanized township, a burial certificate issued by the district hospital was needed for burial or cremation.

#### No formal mandatory notification of death events by households

**Fig. 3:**
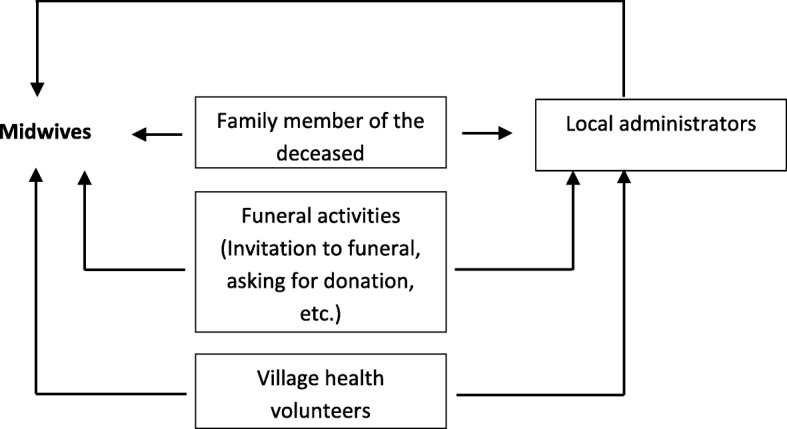
Sources of information of deaths for Midwives (MW)

According to FGDs and KIIs with health staff, family members in both urban and rural areas did not report to the health center when a person died in their households. Instead, local health staff, specifically MWs, had to collect information and complete them in death record (Form 201) and death register (Form 202) by themselves. To perform their assigned function of recording and registering deaths occurred in their geographical catchment areas, MWs searched for the information of death events through various sources (Fig. 3). For example, through health volunteers who reported death events to MWs when MWs came for provisions for outreach immunization or antenatal care; or through local social practices in which the deceased’s family invited the community to the funeral using a community loud speaker, or through posting a board on the street; or through local administrators such as WA, VTA, 10-household or 100-household leaders.

*“If someone dies in the village, the family invited local residents to the religious practices for the funeral using a loud speaker. So, everyone knows there was a death in certain household. Then, we go there to record the event.”* (MW from RHC)

However, if the deceased is a government staff, the family members reported death to the health center because they need a death certificate for applying family pension.

On the other hand, when a death occurred in the household, family members usually informed the local administrator. According to the FGD with community members from rural areas, even if the family members could not inform the administrator by themselves, the village volunteers reported deaths on behalf of the deceased’s family.

The villagers explained that every village had their own cemetery but it is not necessary to present an official approval letter for burial or cremation. They informed administrators and got a “verbal approval” from local administrator to cremate or bury.

In addition, every village in the study area had a volunteer group which was mostly led by local administrators, and the group provided an assistance to the funeral home in many ways. So, by informing the village leaders, the deceased’s family automatically received aids from the volunteer group in arranging funeral processes.

In contrast, participants from urban areas of a more urbanized township said they had to inform the local administrator because they needed approval letters from the administrative office and the municipal office for cremation or burial. However, such practice was not required in urban areas of the less urbanized township.

It seems information of household deaths reached to the local administrators even though there was no an established system, especially in rural areas. Similarly, the means the MW obtained information of death events from local administrators was not systematic which can seriously affect the completeness of death registration.

*“I requested the village leader to tell me if there is a birth or a death in the village as I had to make a report around 20th of every month.”* (MW from RHC)

In some areas, local administrators or volunteers informed voluntarily to the MWs about death events occurred in the area. In some areas, the MWs had to contact them by phone to complete the forms before they prepare a report.

*“In my area, there are hundred-household leaders. I save their phone numbers so that I can contact them if necessary. At the end of each month, I call and ask them who died in their sections, why, how old the person was and other required information.”* (MW from MCH)

All information from FGDs and KIIs confirms there is no formal system of death reporting to health centers from households. Collection of these data by other means can result in incompleteness of death registration.

### The second barrier: technical capacities

#### The absence of proper and regular on-the-job trainings

Midwives, the front line health workers of vital registration, did not receive proper and regular on-the-job trainings on vital registration. Most of them had attended a 45-min lecture on how to fill out the birth and death records as a part of their pre-employment training program. There were few healthcare providers, TMO, LHV and MWs, who had received a comprehensive training for vital registration more than ten years ago. With the absence of well-designed training programs, most of healthcare providers did not have adequate knowledge of rules, regulations and procedures related to death registration which resulted in inadequacy and low quality of recording vital events. Not many healthcare providers knew the registration of vital events was based on the place that the vital events took place, i.e. place of occurrence. In addition, almost all healthcare providers had not seen vital registration manuals published by the CSO in their office.

#### Ineffective COD certification practices for deaths in the communities

In the study townships, the responsible MW had to assign a possible COD on the death record for deaths that occurred in geographical catchment areas under her responsibility. The MWs usually obtained the information from local administrators or family members who witnessed the mortality event. All MWs agreed that it was challenging for them to get the definitive COD, and most sources of information were not well-grounded.

*“It’s hard to get a correct cause of death information if the person dies at home. Usually, we record the cause as provided by the lay informant.”* (HA from RHC)

Some MWs spelt out that they sometimes filled in the name of a disease which they felt to be common in that age group when no one could provide the reliable information.

*“If we don’t know, just name it as hypertension or diabetes if he or she was old. If he was a heavy drinker, I will name it as liver disease.”* (MW from MCH)

However, if the person died after being discharged from the hospital, it was straightforward for the MWs to acquire valid causes of death from medical records or from laboratory or radiological results.

#### The absence of routine data plausibility checks at the local level

Findings from FGDs and KIIs confirmed that the consistency of vital information (i.e. total number of deaths) recorded in the area between the health sector and administrative office was verified on the last week of each month. Inconsistencies were rectified based on local administrative office data, which was regarded as a more reliable source of death information.

*“Every month, the staff from our department, administrative office and immigration office meets together and check the consistency of data, such as how many deaths are recorded in the health department, how many deaths in the administrative office data and the number of population increased or decreased in the immigration office.”* (TMO from the hospital)

Apart from that, there were no established procedures or defined parameters for consistency and plausibility checks for the submitted death registration data. There was also no specific supervision or monitoring of death registration functions, procedures and quality of data.

### The third barrier: societal contexts

#### Poor community awareness and participation in death reporting and registration

Most community members who participated in the FGDs did not know about the requirement for registering deaths such as when, where, how and why they should report and have them registered. Respondents from rural households reported that they had not registered deaths at all. MWs from FGDs also reported that the public was not aware of requirements for death registration, especially in rural areas. Moreover, they said, some family members did not want to provide information to health officials about the deceased. Many health care providers, MOs and MWs, regarded that people did not report deaths to the health center because of the more limited utility of death certificates compared to birth certificates.

“*Concerning birth registration, we have no difficulties because it has its own utility. But there is no usefulness of a death certificate. Even a burial certificate is not required for cremation. In such a situation, people might question why they need to register, what they can do with a death certificate or where it can be used. They may consider that after the person has already died, why it is necessary.”* (TMO from the hospital)

In urban areas, there were some people who had heard about registering deaths and its usefulness for applying for a family survivor pension. In the more urbanized township, where the certificate issued from the health center must be presented for burial or cremation, people reported deaths to the health center.

## Discussion

To our best knowledge, this study is the first qualitative contribution to assess the challenges of poorly performed VRS in Myanmar, after its quantitative assessment [6, 7]. This qualitative study highlights certain administrative challenges, limited technical capacities and societal ignorance. These bottle necks have to be resolved in order to improve the performance of death registration.

The findings identified challenges of death registration operations in two different settings: a more urbanized township and a less urbanized township. The basic principles and management of death registration and how it is operated are similar; death notifications are informally managed, and a death or burial certificate is not strictly required in rural areas. While district hospitals issue burial certificates in the city in the more urbanized township, these certificates are provided by Township General Administrative Office (TGAO) or Development Affairs Office (DAO) in the town of the less urbanized township.

While a strong and comprehensive legal framework is critically important for completeness and accuracy of vital registration data [13], the country is absent of a specific vital registration law. Neither the related laws applied for death reporting nor the enforcement is adequate. In the current situation, while the law specifies to report deaths to administrators, health staffs who work closely in the community are assigned to ensure that all births and deaths are registered, recorded and reported to the Central Statistical Organization (CSO).

It is essential for Myanmar to legislate a law defining a vital registration system that requires mandatory registration and reporting of all births and deaths to health facilities. In addition, the law, rules and regulations governing the VRS should cover all essential elements which support a comprehensive legal framework, in particular penalty for non-compliance, as described in details in subcomponent A1 “National legal framework for civil registration and vital statistics systems” of the World Health Organization assessment framework [14].

Death registration practices and procedures were slightly different between two study townships as well as between urban and rural areas. The major difference is the need for a burial certificate. Inconsistent procedures and limited application of death or burial certificates, especially in rural areas, hinders the system to run effectively. In Thailand where a death certificate must be presented for cremation, death registration was almost universal with a 98.4% completeness [15]. Death notification by households within 24 h is mandatory in Thailand, while not more than a 1000 baht (approximately $32) fine for notification beyond 24 h is strictly enforced [16]. To improve the completeness of the country’s VRS, it is strongly recommended to create the value, the importance and usefulness of a death/burial certificate such as for approval of cremation and burial in both urban and rural areas and with penalty for non-compliance. In Thailand, birth registration is importance for citizen’s entitlements to healthcare in the context of universal health coverage, and other social welfare.

In the current situation, the midwives (MWs) have to collect vital information from different sources, sometimes in an ad hoc manner by themselves rather than mandatory death notification by household members. In the Myanmar health care system, MWs have multiple tasks including vital registration, apart from their primary duty such as antenatal care, delivery and post-natal care. When completeness of registered deaths largely depends on the performance of the assigned MW, their performance on registering vital events would probably decline if they handle an increased health service workload. The absence of effective and mandatory formal death notification by households leads to an increased burden for MWs and jeopardizes the completeness of vital events.

The followings can reinforce registration functions of the health department as well as to reassure higher completeness, correctness and accuracy of mortality information. For example, work together among the focal department (i.e. CSO), the implementing organization (i.e. health centers) and partner organization (i.e. TGAO) in mobilizing public awareness on the importance of notification of vital events; provide the general public with clear information and instructions about the procedure and requirements of reporting all deaths to the health centers; establish strong and systematic links among related organizations, such as health sector, TGAO and the Police Force, to ensure information of all deaths in the community reach health centers; initiate mobile registration services in remote and hard-to-reach areas with limited accessibility to health centers; and ensure the size of population and number of wards or villages assigned to MWs are reasonable to perform their functions effectively.

The availability of knowledgeable well-trained staff is an essential prerequisite for vital registration [17]. However, the healthcare providers in the study townships did not have adequate knowledge of vital registration, particularly death registration, as a consequence of the absence of regular refresher trainings and unavailability of the manuals or guidelines in their offices. Provision of regular trainings and refresher trainings to the vital registration staff will contribute significantly to the strengthening of the VRS. The refresher training is a good opportunity for updating and solving their problems encountered in the field [17]. In South Africa for example, the strategies applied included distribution of manuals, flow charts and standard operating procedures on birth and death registration to health staff, resulting in substantial improvement in completeness of death registration in less than two decades [18]. A well-designed training program which covers the essential elements needed for operating an effective VRS based on the needs of the target group according to the UN guidance [19] needs to be developed, and training materials and guidelines should be able to provide to each and every staff who are performing vital registration functions. Training of new cadres of staffs and refresher courses to in-service staffs should be made mandatory.

In a country where more than 80% of deaths are occurring outside health facilities [6], COD certification practice by health workers for deaths occurring in the community produces unreliable and inaccurate diagnosis or a high proportion of ill-defined COD. To overcome these challenges, the application of a standard verbal autopsy tool should be introduced and up-scaled [20]. As a strategy to improve the quality of COD, the automated verbal autopsy platform (SmartVA), as part of the CRVS Data for Health Initiatives [21], was introduced in some selected townships of Myanmar since 2016 to identify COD for community deaths. In the SmartVA system, data collection is conducted by basic health staff, such as MWs, using electronic questionnaires on hand-held tablet, and systematic algorithms was applied to estimate probable COD. In Myanmar, the SmartVA has yet to upscale and assess its contribution to the improvement of the quality of COD estimation. Encouragingly, some countries, such as Bangladesh and Sri Lanka, have got positive experiences regarding introducing SmartVA; the system produced reliable and quality COD among community deaths [22, 23].

Though routine data plausibility checks and specific supervision are integral parts of the VRS [24], there were no such activities in the study townships. A simple and cost effective solution, the simple consistency check by calculating rates and comparing them with rates from other available sources and estimating the proportion of unknown COD, can be applied on a routine basis at the local level [24]. Guidelines and standard operating procedures for routine data verification of consistency and plausibility should be provided to staffs from health facilities responsible for supervision of recording, registering and reporting of vital events.

In addition to the low level of public awareness, the limited perceived benefit of death registration has led to poor adherence to mandatory death notification. This challenge is not uncommon in other developing countries [25, 26]. Linking death notification with certain social welfare or benefits, such as a small funeral financial support, together with activities of increasing community awareness can improve the completeness of death registration.

In responses to the reporting requirement of SDG (Sustainable Development Goals) Indicator 17.19.2 [the proportion of countries that (a) have conducted at least one population and housing census in the last 10 years; and (b) have achieved 100% birth registration and 80% death registration] [27], the Myanmar government needs to fully commit and invest in the VRS. Allocation of adequate financial resources is obligatory to implement and sustain a well-functioning VRS. Allocation of adequate financial resources is needed in the areas of provision and maintenance of sufficient registration facilities in health centers such as registration forms, stationary, telephones, photo copiers, computer, internet; regular delivery of good quality trainings to health staff; dissemination of updated training materials and guidelines; establishing mobile registration facilities for health centers from hard-to-reach areas; and conducting research to identify the most effective implementation strategy and to assess implementation barriers and possible solutions; and scaling up the SmartVA platform. Investment in the VRS will improve the reliability of vital statistics that can be used for efficient resource allocation in the health system [24].

There were some limitations in the study. Firstly, as data collection was conducted only in two townships, the findings should not be over-generalized. Generally, the findings could explain the situation related to death registration currently operating in the country. However, the two townships do not represent death registration operations in well-developed cities with higher accessibility to services; or the least developed areas where there are unfavorable geographical and socio-economic conditions. Secondly, the study explored only the barriers existed at the operational level, i.e. data collection, registering and reporting, but not at the central level, which takes central role in data coding, processing and publishing mortality statistics. As a future action, a comprehensive assessment of the CRVS system as a whole, including inputs, processes and outputs of the system, is recommended by using the WHO guidance tool [14]. Table 3 provides a synthesis of recommendations.

**Table 3:** Recommendations for the improvement of death registration in Myanmar

Recommendations for each area	
Legal enforcement and administrative support	
• Enforce the existing vital registration related laws • Develop a comprehensive legal framework including necessary elements in the VRS in line with the World Health Organization (WHO) assessment framework of the five key components of the Civil Registration and Vital Statistics systems • Regulate the mandatory requirement of a burial certificate, as a condition to gain an approval for burial or cremation in urban and rural areas • Establish mandatory household death notification to health centers and effective enforcement • Link death notification with some social services or benefits to incentivize household compliance	
Technical capacity strengthening	
• Provide regular on-the-job trainings on vital registration and cause-of-death certification to healthcare providers • Make available of working manuals, flowcharts and guidelines, which includes clear and adequate instructions, to all healthcare providers • Establish a routine practice of standard data consistency and plausibility checks at the local level • Upscale SmartVA platform for improved accuracy of causes-of-death	
Societal awareness and engagement	
• Raise public awareness about the importance of death notification, and mandatory requirement of death registration through social mobilization	
Others	
• Perform a comprehensive assessment of the CRVS system by using the WHO guidance tool • Ensure adequate resource allocation to the health sector to be able to perform vital registration functions efficiently at operational level	

## Conclusions

The study indicated weaknesses in the current death registration at the operational level, in the areas of administration, technical capacities and public cooperation, which are valuable information for the implementing organizations in making effort to strengthen death registration in Myanmar.

## Additional files

Additional file 1: Key Informant Interview Guide-1. Guide for Key Informant Interview with Medical Officers and Health Assistants. (DOCX 19 kb)

Additional file 2: Key Informant Interview Guide-2. Guide for Key Informant Interview with Local Administrators. (DOCX 17 kb)

Additional file 3: Focus Group Discussion Guide-1. Guide for Focus Group Discussion with Lady Health Visitors and Midwives. (DOCX 18 kb)

Additional file 4: Focus Group Discussion Guide-2. Guide for Focus Group Discussion with Community Members (DOCX 18 kb)

## Abbreviations

COD: Cause of Death
CSO: Central Statistical Organization
DAO: Developmental Affairs Office
DMS: Department of Medical Services
DOPH: Department of Public Health
FGD: Focus Group Discussion
GAD: General Administrative Department
HA: Health Assistant
KII: Key Informant Interview
LHV: Lady Health Visitor
MO: Medical Officer
MW: Midwife
SDG: Sustainable Development Goals
TGAO: Township General Administrative Office
TMO: Township Medical Officer
UN: United Nations
UNICEF: United Nations International Children’s Emergency Fund
VRS: Vital Registration System
VSPI: Vital Statistics Performance Index
VTA: Village Tract Administrator
WA: Ward Administrator
WHO: World Health Organization
