# Supplementary material for: Reliable mortality statistics in Myanmar: a qualitative assessment of challenges in two townships
Source: BMC Public Health. 2019 Mar 29;19:356. doi: 10.1186/s12889-019-6671-y (PMC6441185; doi:10.1186/s12889-019-6671-y)
Supplement: Supplementary file 1 — Key Informant Interview Guide-1. Guide for Key Informant Interview with Medical Officers and Health Assistants. (DOCX 19 kb) [file 12889_2019_6671_MOESM1_ESM.docx]

**Key Informant Interview Guide-1**

**Guide for Key Informant Interview with Medical Officers and Health Assistants**

1. **Background characteristics**
2. Age, Sex, Residence, Type of health facility currently working,
3. Working experience (years in service), Duration of living in current residence
4. **Death registration in the VRS**
5. Are there any laws or legislations which are applied for registration of deaths in this area? What are they? Are they practicing in real situations?
   1. Presence of legislation that states death registration is compulsory
   2. Presence of regulations to report all death events to vital statistics system within a given time
   3. Presence of legislation that states death has to be certified by cause and who can certify the cause of death
6. Do you think health facilities in this area have capacities to carry out death registration functions efficiently? Why? What are the necessities?
7. Availability of facilities to carry out necessary functions (e.g. offices, forms, papers, books, telephones)
8. Have your health staff ever received trainings to carry out necessary functions (to whom, how often)
9. Availability of skilled personals
10. In the VRS, are there any collaborations between health department and other departments or organization in death registration operations? What are they? How are they working?
11. Could you please explain in detail how death registration system is functioning in your area? Registration of deaths at health facility (or) at home?
12. Who contributes in registration of deaths in this health facility? How do they contribute?
13. How death-records are reported from lower level to higher level (from local to regional to central office)? How they make ensure to report deaths within agreed times?
14. How death certification is performed within and outside hospitals? How is it different within and outside hospital?
15. Use of standard international form of medical certificate of cause of death for reporting
16. Any established evaluation method for completeness of registration of deaths in this facility? If yes, how? By whom? Frequency?
17. Most recent evaluation to assess completeness of death registration
18. How do you make sure to obtain quality cause-of-death data in this area?
19. Training for doctors for certifying cause of death
20. Are there any routine data quality and plausibility check for death registration data? If yes, how?
21. Practice of consistency and plausibility checks on mortality level and cause of death
22. Any supervision in the VRS? Any regular report or meeting for the functions of vital registration?
23. **Perceptions on public awareness and practice on death registration**
24. What do you think about awareness, knowledge, attitude and practice of people towards registering deaths in your area? Why?
25. **Suggestions to improve the death registration in the area**
26. What are the problems for you or your staff in recording and registering deaths, issuing certificates and reporting of deaths?
