# Supplementary material for: Reliable mortality statistics in Myanmar: a qualitative assessment of challenges in two townships
Source: BMC Public Health. 2019 Mar 29;19:356. doi: 10.1186/s12889-019-6671-y (PMC6441185; doi:10.1186/s12889-019-6671-y)
Supplement: Supplementary file 2 — Key Informant Interview Guide-2. Guide for Key Informant Interview with Local Administrators. (DOCX 17 kb) [file 12889_2019_6671_MOESM2_ESM.docx]

**Key Informant Interview Guide-2**

**Guide for Key Informant Interview with Local Administrators**

1. **Background characteristics**
2. Age, Sex, Position,
3. Name of ward or village tract or village
4. Working experience as a local administrator
5. **Death recording, reporting**
6. How does your office (administrative office) do for death recording and reporting? (How is recording and reporting? Where to report? When? How frequent?)
7. What is your role in death recording and reporting?
8. Please share with us your death recording and reporting experiences.
9. What challenges do you face in death recording and reporting?
10. **Linkage with health sector for information of vital events**
11. Do you have any linkage with health facilities in your area to share information of vital events? How does it go on?
12. Are there any problems when you work with health sector for sharing vital information?
13. **Perceptions on public awareness and practice on death registration**
14. What do you think about public awareness and participation in death registration?
15. **Suggestions to improve the death registration in the area**
16. How do you think we can improve death reporting and registration?
