# Supplementary material for: Reliable mortality statistics in Myanmar: a qualitative assessment of challenges in two townships
Source: BMC Public Health. 2019 Mar 29;19:356. doi: 10.1186/s12889-019-6671-y (PMC6441185; doi:10.1186/s12889-019-6671-y)
Supplement: Supplementary file 3 — Focus Group Discussion Guide-1. Guide for Focus Group Discussion with Lady Health Visitors and Midwives. (DOCX 18 kb) [file 12889_2019_6671_MOESM3_ESM.docx]

**Focus Group Discussion Guide-1**

**Guide for Focus Group Discussion with Lady Health Visitors and Midwives**

1. **Background information of respondents**
2. Age, Sex, Occupation, Permanent residence, Type of health facility concerned
3. Duration of living in current residence, Duration of working experience
4. **Death registration in the study area**
5. How death registration is operating in this area? (Are there any organizations or departments involved in the VRS apart from the health sector? How do they involve?)
6. What are the functions of basic health staff in the death registration?
7. Have you received training about registration, recording and reporting of vital events? How many times? What issues are involved in the training? Have you received any guideline or SOP (Standard Operating Procedure) for the VRS?
8. Does the government or the township authority provide any facilities for your functions in your assigned area, including registration of vital events? What are they? What kind of facilities do you need?
9. Who can report/register a death? Where people can report/register deaths? When people have to report/register deaths? Is there any regulation which states that a household member has to report death to the health center within a specified period? What will happen if it is late registration? Which documents and information are necessary for registering deaths? Who is authorized to issue a death certificate? Who is authorized to certify the cause of death?
10. Please explain the procedure of registration of deaths, verifying and reporting data in this area?
11. What do you do if people do not report/register a death event which occurred in their household? Why do you think people do not report or register a death event?
12. Are there any groups (i.e. non-governmental groups or voluntary groups) in this area that provide services to households which had a death event? Do their works help to improve registration of deaths? How?
13. Is there any supervision for registration, recording and reporting of vital events? From whom? How? Frequency? What do they do in supervision?
14. Are there any routine data quality and plausibility check for death registration data? If yes, how? (Practice of consistency and plausibility checks on mortality level and cause of death)
15. Could you please discuss about the problems you encountered during the process of registration, verifying and reporting of death events? (Administrative problems, technical problems and social problems)
16. **Perceptions on public awareness and practice on death registration**
17. How do you think about people’s awareness, knowledge and attitude towards death registration? Why?
18. **Suggestions to improve the death registration in the area**
19. What are your suggestions to improve registration of deaths in your area?
