# Supplementary material for: Reliable mortality statistics in Myanmar: a qualitative assessment of challenges in two townships
Source: BMC Public Health. 2019 Mar 29;19:356. doi: 10.1186/s12889-019-6671-y (PMC6441185; doi:10.1186/s12889-019-6671-y)
Supplement: Supplementary file 4 — Focus Group Discussion Guide-2. Guide for Focus Group Discussion with Community Members (DOCX 18 kb) [file 12889_2019_6671_MOESM4_ESM.docx]

**Focus Group Discussion Guide-2**

**Guide for Focus Group Discussion with Community Members**

1. **Background characteristics**
2. Age, Sex, Education level, Occupation, Residence (Rural/Urban)
3. Duration of living in current residence (years)
4. **Opinions and experiences of the public about death registration**
5. What do people in your area do when a death event occurs in their households? Report of death? To where? To whom? By whom? How?
6. Do you think people in your area usually report deaths to health centers? Why?
7. Could you all please share your experiences in reporting or registering the death event? Any problems or difficulties? (Ask those who registered the death event occurred in their households)
8. What do you think about the *registration procedure* in your area? (Simple or Complicated? Why do you think so?)
9. Did you request death or burial certificate when the death event occurred in the household?
   - - If no, why?
     - If yes, why? How?
10. Which circumstances do you think can encourage/discourage people reporting/ registering deaths?
11. **Suggestions to improve death registration in the area**
12. Tell me about your opinions on how registration of deaths can be made better in your area?
